# Crucial amino acids identified in Δ12 fatty acid desaturases related to linoleic acid production in *Perilla frutescens*

**DOI:** 10.3389/fpls.2024.1464388

**Published:** 2024-09-10

**Authors:** Zhenke Wu, Mingkai Li, Xiqin Liang, Jun Wang, Guoli Wang, Qi Shen, Tianyue An

**Affiliations:** ^1^ Featured Laboratory for Biosynthesis and Target Discovery of Active Components of Traditional Chinese Medicine, School of Traditional Chinese Medicine, Binzhou Medical University, Yantai, China; ^2^ Institute of Medical Plant Physiology and Ecology, School of Pharmaceutical Sciences, Guangzhou University of Chinese Medicine, Guangzhou, China

**Keywords:** *Perilla frutescens*, catalytic activity, molecular docking, mutation, FAD2 desaturase

## Abstract

Perilla oil from the medicinal crop *Perilla frutescens* possess a wide range of biological activities and is generally used as an edible oil in many countries. The molecular basis for its formation is of particular relevance to perilla and its breeders. Here in the present study, four *PfFAD2* genes were identified in different perilla cultivars, PF40 and PF70, with distinct oil content levels, respectively. Their function was characterized in engineered yeast strain, and among them, PfFAD2-1^PF40^, PfFAD2-1^PF70^ had no LA biosynthesis ability, while PfFAD2-2^PF40^ in cultivar with high oil content levels possessed higher catalytic activity than PfFAD2-2^PF70^. Key amino acid residues responsible for the enhanced catalytic activity of PfFAD2-2^PF40^ was identified as residue R221 through sequence alignment, molecular docking, and site-directed mutation studies. Moreover, another four amino acid residues influencing PfFAD2 catalytic activity were discovered through random mutation analysis. This study lays a theoretical foundation for the genetic improvement of high-oil-content perilla cultivars and the biosynthesis of LA and its derivatives.

## 1 Introduction

Linoleic acid (LA) is an ω-6 polyunsaturated fatty acid (FA) and is indispensable for human health (Belury, 2023; Saini and Keum, 2018). LA is pivotal in health supplements and food products, and exhibits notable pharmacological activities, such as antioxidant and anti-inflammatory effects. Its applications extend to metabolic regulation, blood sugar management, and cardiovascular disease prevention (Yang et al., 2020; Alarcon-Gil et al., 2022; Hamilton and Klett, 2021; Marangoni et al., 2020). Furthermore, LA serves as a vital precursor for α-linolenic acid synthesis, endowing it with considerable economic significance and development potential (Choudhary and Mishra, 2021). Currently, large-scale LA production is predominantly achieved through extraction from natural oilseed crops. Consequently, breeding these crops is essential for the sustainable advancement of the LA synthesis industry (Sana et al., 2018; Kanbar et al., 2023; Yang et al., 2023; Pathak et al., 2014). Perilla seeds, harvested from the medicinal crop *Perilla frutescens*, are rich in unsaturated fatty acids, including oleic acid (OA), LA, and α-linolenic acid, rendering them a significant oilseed crop (Kim et al., 2020). Nonetheless, the diverse perilla cultivars and the varying LA content among them constrain the large-scale cultivation and improvement of these varieties (Park et al., 2022; Lee et al., 2019). Therefore, investigating the genetic basis of LA phenotypes and identifying Δ12 fatty acid desaturases (FAD2) with high catalytic efficiency for LA synthesis is crucial for perilla breeding.

Omics technologies offer an efficient approach to studying cellular phenotypes and gene functions comprehensively (Fagerberg et al., 2014). Through omics sequencing and bioinformatics analysis, plant genes can be scrutinized to pinpoint relevant functional genes and elucidate the molecular mechanisms underlying specific biological processes (Su et al., 2023). Consequently, high-throughput sequencing technologies, known for their substantial data output and accuracy, are extensively employed to analyze secondary metabolite variations between plant varieties and to identify key enzymes. For example, transcriptome sequencing analysis of different *Ziziphus jujuba* Mill. varieties can identify key genes in the flavonoid biosynthesis pathway and elucidate the molecular mechanisms affecting epicatechin content across different varieties (Wang et al., 2023). Similarly, transcriptomic and phenotypic analyses of differential genes associated with unsaturated fatty acid content in soybeans can provide insights into enhancing fatty acid composition in soybean seeds (Liu et al., 2022a).

The eukaryotic model organism *Saccharomyces cerevisiae*, renowned for its well-characterized genetic background and ease of genetic manipulation, is widely utilized for enzyme functional verification, including terpene synthases and fatty acid desaturases (Deng et al., 2022; Runguphan and Keasling, 2014). Yeast cells possess a natural fatty acid synthesis pathway capable of generating pyruvate through glycolysis, which subsequently undergoes decarboxylation, dehydrogenation, and a series of reactions to produce malonyl-CoA. Using malonyl-CoA as a basic unit, yeast cells can synthesize various free fatty acids (FAs), among which OA, a monounsaturated fatty acid, serves as a crucial precursor for LA synthesis. Consequently, employing OA-overproducing *S. cerevisiae* strains for the functional characterization of fatty acid desaturases is currently an effective strategy (Kuziora et al., 1983; Miao et al., 2019). Furthermore, *S. cerevisiae* is extensively applied in the construction of microbial cell factories for fatty acids and their derivatives (Guo et al., 2022).

Previous research has reported the genome data of perilla and conducted transcriptome analyses on various perilla cultivars (Wu et al., 2021). In this study, two perilla cultivars with distinct oil content levels, PF40 and PF70, were selected (Zou et al., 2024). Four *PfFAD2* genes were identified from their transcriptome data and designated as PfFAD2-1^PF40^, PfFAD2-1^PF70^, PfFAD2-2^PF40^ and PfFAD2-2^PF70^, respectively. Then these genes were functionally characterized using our constructed OA-overproducing *S. cerevisiae* strain. The results revealed that PfFAD2-1^PF40^ and PfFAD2-1^PF70^ lacked the capability to synthesize LA, while only PfFAD2-2^PF40^ and PfFAD2-2^PF70^ could catalyze the conversion of OA to LA. Furthermore, PfFAD2-2^PF40^, derived from the high oil-producing perilla cultivar, exhibited superior catalytic activity. Through sequence alignment, molecular docking, and site-directed mutation studies, key amino acid residue responsible for the enhanced catalytic activity of PfFAD2-2^PF40^ compared to PfFAD2-2^PF70^ were identified. Additional amino acid residues influencing PfFAD2 catalytic activity were discovered through random mutation analysis. This study lays a theoretical foundation for the genetic improvement of high-oil-content perilla cultivars and provides optimal microbial elements for the biosynthesis of LA and its derivatives.

## 2 Materials and methods

### 2.1 Plant materials and yeast strains

Perilla seeds were collected from their natural habitat, specifically the plant material cultivation greenhouse of Guangzhou University of Chinese Medicine, during their developmental period (late August to mid-September). Seeds from various perilla cultivars were harvested within one-month post-flowering, with calyxes removed before storage in liquid nitrogen for future use. The *S. cerevisiae* strain BY4741, used as the chassis strain in this study, was purchased from Shanghai Yuanye Bio-Technology Co., Ltd.

### 2.2 Gene cloning

RNA was extracted from perilla seeds using the FastPure Plant Total RNA Isolation Kit (Vazyme, China). Subsequently, 1 μL of RNA was reverse transcribed into cDNA using the HiScript III 1st Strand cDNA Synthesis Kit (+gDNA wiper) (Vazyme, China). Following the manufacturer’s protocol, the full-length sequence of the target gene was cloned, ligated into a Blunt vector (TransGen Biotech, China), and sequenced. Primers used in this part were listed in **Supplementary** **Table S1**.

### 2.3 Construction of engineered yeast strain

Firstly, the GAL10p and GAL1p promoters of pESC-URA/HIS/LEU (Miaoling Biology, China) were replaced by TEF1p and PGK1p to get glucose induced vector pTP-URA/HIS/LEU. *TGL1*, *TGL3* and *TGL5* were cloned from the genomic DNA of *S. cerevisiae* strain BY4741. *TGL1* and *TGL5* were ligated into pTP-URA, and *TGL3* was ligated into pTP-HIS. Then the expression cassette of URA maker and *TGL1* and *TGL5*, and the expression cassette of HIS maker and *TGL3*, were cloned from the constructed pTP vectors. These two expression cassettes were integrated into the *FAA1* and *FAA4* sites in the yeast genome by using the method reported in the previous work (Jiang et al., 2022). The schematic diagram illustrating the construction of the yeast strain was shown in **Supplementary Figure S1**. Primers used in this part were listed in **Supplementary** **Table S1**.

### 2.4 Construction and transformation of yeast expression vectors

The cloned *PfFAD2* gene was inserted into the yeast expression vector pTP-LEU between the Sac I and Not I restriction sites in the multiple cloning sites, and verified by sequencing. The transformation plasmid was mixed with yeast competent cells in a solution containing 50% PEG3350 (Solarbio, China), 1 mol/L lithium chloride (Solarbio, China), and salmon sperm DNA (Solarbio, China), followed by incubation at 42°C for 1 h. The transformed yeast cells were centrifuged at 9500 rpm for 1 min, and the supernatant was discarded. The cell pellet was resuspended in YPD medium and cultured at 30°C with shaking at 220 rpm for 3 h. After washed twice with distilled water, the samples were cultured on appropriately labeled solid media. Primers used in this part were listed in **Supplementary** **Table S1**.

### 2.5 Shake flask cultivation

A single colony of positive yeast strains was cultured in 5 mL of SD medium (6.67 g/L yeast nitrogen base without amino acids (BD Difco, America), 20 g/L glucose (Macklin, China), supplemented with corresponding amino acids) at 30°C and 220 rpm. Upon reaching an OD_600_ of 1.5-2.5, an appropriate volume of the seed culture was transferred to 50 mL of induction medium (6.67 g/L yeast nitrogen source without amino acids, 20 g/L glucose, supplemented with appropriate amino acids). The culture was then incubated at 30°C and 220 rpm for 5 days.

### 2.6 Extraction and detection of compounds

After fermentation, 600 μL of the cultured broth was collected and combined with 30 μL of 40% tetrabutylammonium hydroxide (Macklin, China) and 200 μL of dichloromethane (Macklin, China) solution containing 200 mM methyl iodide (Sigma-Aldrich, America). The mixture was vortexed at 2000 rpm for 30 min and then centrifuged at 5000 g. The lower dichloromethane layer was transferred to a glass vial, dried, and resuspended in 200 μL of n-hexane. The prepared sample was subsequently analyzed by gas chromatography-mass spectrometry (GC-MS).

GC-MS analysis was conducted using an Agilent 7890 gas chromatograph equipped with an HP-5MS column and a Saturn 2100 ion trap mass spectrometer. Samples were injected in splitless mode with an injection volume of 1 μL. The temperature program was as follows: initial temperature of 50°C held for 2 min, increased to 140°C at a rate of 30°C/min, then raised to 280°C at a rate of 10°C/min, and held for 3 min.

### 2.7 Site-directed and random mutation

The selected *PfFAD2-2^PF70^* containing yeast expression vector served as a template to generate the *PfFAD2-2^PF70^* mutants using the Mut Express II Fast Mutagenesis Kit V2 (Vazyme, China). The amplified plasmid underwent digestion with the restriction endonuclease DpnI (Thermo Fisher Scientific, America) for one hour, followed by transformation into competent *Escherichia coli* DH5α cells ((TransGen Biotech, China)). Positive colonies were identified and sequenced to confirm the site-directed mutation. Primers used in this part were listed in **Supplementary** **Table S1**.

Random mutation was conducted using the *PfFAD2-2^PF40^* gene as a template. The fragment was amplified with the Instant Error-prone PCR Kit. Subsequently, the amplified fragment was ligated into the pTP-LEU vector, and positive *E. coli* DH5α colonies were selected for sequencing. Primers used in this part were listed in **Supplementary** **Table S1**.

### 2.8 Statistical analysis

The data analysis was done by using GraphPad Prism 8 software. The significant differences were performed by unpaired two-tailed Student’s t-tests. P-value < 0.05 was considered as statistically significant. The experiments were performed in triplicate, and the data was presented as the mean ± standard deviation (SD).

## 3 Results

### 3.1 Cloning and functional analysis of PfFAD2 from different perilla cultivars

**Figure 1:**
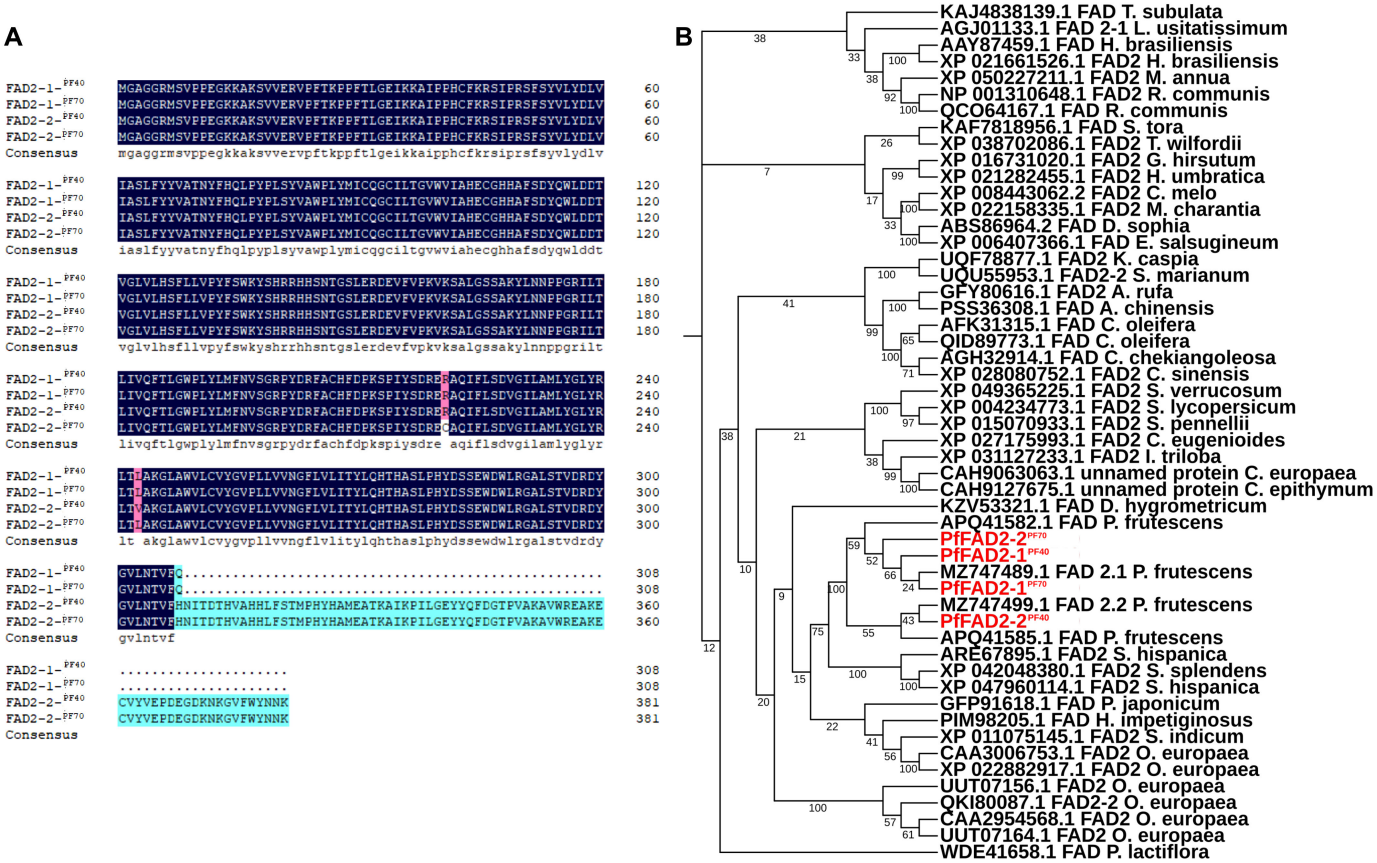
The sequence alignment and phylogenetic analysis of the cloned PfFAD2. **(A)** The sequence alignment of different PfFAD2 in PF40 and PF70 cultivars. The amino acids in blue color and cyan were identical, and the positions of different amino acids were shown in in purple color. **(B)** The phylogenetic analysis of PfFAD2.

Our previous study has found that the oil contents in the seeds of perilla cultivars PF40 and PF70 was different, and 343.51 mg/g and 281.34 mg/g of total oil contents were detected in these two cultivars, respectively (Zou et al., 2024). Meanwhile, the LA content in PF40 was higher than that in PF70 (Zou et al., 2024). By blasting the genome and transcriptome data of perilla using the previously identified *P. frutescens* fatty acid desaturase 2.1 (GenBank accession No. MZ747489.1) and 2.2 (GenBank accession No. MZ747499.1) as query, a total of four *FAD2* transcripts were identified from the high oil-producing perilla cultivar PF40 and the low oil-content perilla cultivar PF70. Their full-length gene sequences were cloned using 5’/3’-RACE PCR and designated as *PfFAD2-1^PF40^*, *PfFAD2-1^PF70^*, *PfFAD2-2^PF40^* and *PfFAD2-2^PF70^*. The full length of *PfFAD2-1^PF40^* and *PfFAD2-1^PF70^* are 927 bp, encoding a protein of 309 aa, while *PfFAD2-2^PF40^* and *PfFAD2-2^PF70^* are 1149 bp long, encoding a protein of 383 aa. Protein sequence alignment revealed that the amino acid sequences of PfFAD2-1^PF40^ and PfFAD2-1^PF70^ are identical, whereas PfFAD2-2^PF40^ and PfFAD2-2^PF70^ differ in two amino acids, located at positions 243 and 221 (**Figure 1A**). To preliminarily explore the function of these cloned genes, we screened for identical or similar sequences from different species by Protein BLAST program. Then multiple sequence alignment was performed on the selected sequences and cloned genes, and the phylogenetic tree was constructed by MEGA software (Tamura et al., 2021). The phylogenetic analysis indicated that all four genes were predicted to encode Δ12 fatty acid desaturases (**Figure 1B**).

### 3.2 Functional characterization of PfFAD2

OA, which constitutes approximately 80% of free FAs in the cytoplasm of *S. cerevisiae*, serves as the precursor for LA, indicating that intracellular free FA level is the primary limiting factor for LA synthesis (Klug and Daum, 2014). Previous research has demonstrated that lipid droplets in *S. cerevisiae* generate free FAs through the activity of steryl ester hydrolase Tgl1 and TAG lipases Tgl3 and Tgl5 (Athenstaedt and Daum, 2005). Additionally, free FAs within the cytoplasm of *S. cerevisiae* can re-enter the β-oxidation pathway catalyzed by fatty acyl-CoA synthetases Faa1 and Faa4, resulting in OA consumption (Leber et al., 2015).

**Figure 2:**
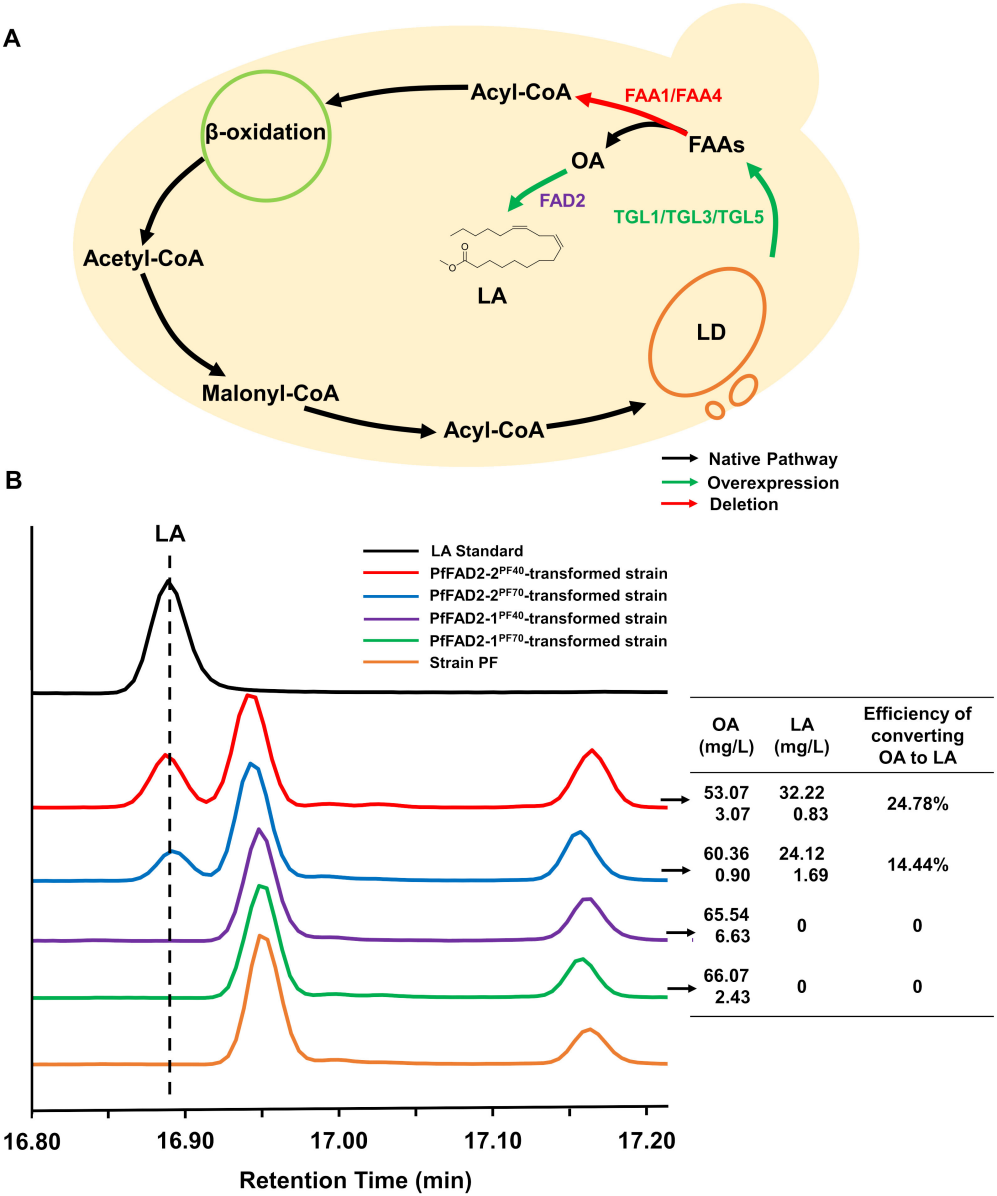
The functional characterization of PfFAD2 by yeast expression system. **(A)** The metabolic engineering of yeast for the accumulation of OA. The green color represented the overexpressed enzymes and the red color represented the deleted enzymes. **(B)** The detection and contents of OA and LA in different yeast strains. The left represented the GC profiles of LA standard and different engineered yeast strains, and the right represented the contents of OA, LA and the efficiency of converting oleic acid to LA in the four engineered yeast strains carrying different PfFAD2 enzymes.

Here, the *S. cerevisiae* strain was employed as a chassis for the functional verification of the cloned *PfFAD2* genes. Due to the relatively low OA content, the substrate for PfFAD2, in *S. cerevisiae*, metabolic engineering optimization was performed on *S. cerevisiae* BY4741 to ensure an adequate supply of OA as a precursor for PfFAD2. This was achieved by overexpressing *TGL1*, *TGL3*, and *TGL5* to promote the release of free FAs from lipid droplets and by knocking out *FAA1* and *FAA4* to reduce the activation of free FAs (**Figure 2A**). Consequently, according to the standard curve of OA (**Supplementary Figure S2**), the resulting engineered strain PF produced 70.55 ± 6.27 mg/L of OA, rendering it suitable as a chassis strain for FAD2 functional verification. Subsequently, the four cloned *PfFAD2* genes were individually constructed into the yeast expression vector pTP-LEU and transformed into the aforementioned engineered yeast strain PF. After fermentation, the target products were detected by using GC-MS equipment. By comparing the retention times of GC peaks and MS spectra with standards (**Supplementary Figure S3**), LA was detected in strains transformed with plasmids carrying *PfFAD2-2^PF40^* and *PfFAD2-2^PF70^* genes (**Figure 2B**). Conversely, no LA production was observed in strains transformed with *PfFAD2-1^PF40^* and *PfFAD2-1^PF70^* contained plasmids (**Figure 2B**). This indicates that *PfFAD2-2^PF40^* and *PfFAD2-2^PF70^* can catalyze LA production, while *PfFAD2-1^PF40^* and *PfFAD2-1^PF70^* lack this catalytic ability. Previous studies have shown that the deletion of consecutive amino acids near the active site can lead to reduced enzyme activity, whereas the deletion of consecutive amino acids at the C-terminus can result in a complete loss of enzyme activity (Arjomand et al., 2016; Zhu et al., 2016). Therefore, it is hypothesized that the functional loss of PfFAD2-1^PF40^ and PfFAD2-1^PF70^ may be related to the absence of a highly relevant functional region at its C-terminus associated with LA synthase catalytic function (**Figure 1A**).

### 3.3 Key amino acids residues affecting the catalytic ability of PfFAD2-2 in different perilla cultivars

**Figure 3:**
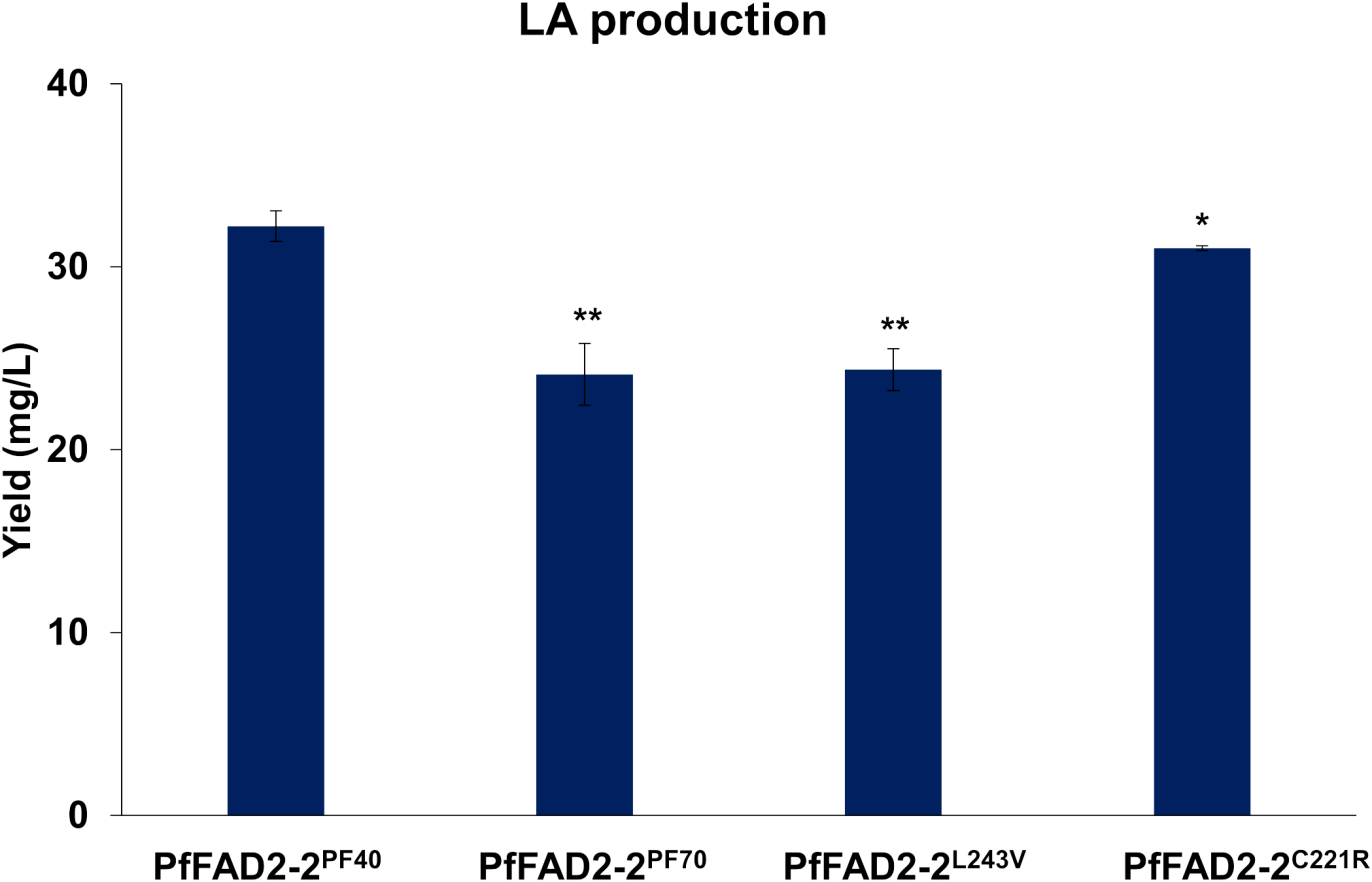
The LA production in yeast strains with different PfFAD2 transformation. PfFAD2-2^L243V^ and PfFAD2-2^C221R^ were mutated from PfFAD2-2^PF70^. The asterisks indicate significant differences (**p < 0.01, *p < 0.05).

During the functional characterization of PfFAD2-2, it was observed that PfFAD2-2^PF40^, derived from the high oil-producing perilla cultivar PF40, catalyzed a higher yield of LA (32.22 ± 0.83 mg/L) compared to PfFAD2-2^PF70^ (24.12 ± 1.69 mg/L) from the low oil-producing cultivar PF70 (**Figure 3**). This suggests that PfFAD2-2^PF40^ possesses higher catalytic ability. This result was consistent with the finding that PF40 contained more LA than PF70 (Zou et al., 2024). Sequence alignment previously revealed two residues’ differences between PfFAD2-2^PF40^ and PfFAD2-2^PF70^ protein sequences, located at positions 221 and 243 (**Figure 1A**). To investigate whether these two sites were responsible for the difference in catalytic ability, the two amino acid sites in the PfFAD2-2^PF70^ were mutated to match those of PfFAD2-2^PF40^, resulting in two variants: PfFAD2-2^L243V^ and PfFAD2-2^C221R^. These variants were then transformed into the engineered yeast strain to measure LA production. Results indicated that the strain transformed with PfFAD2-2^L243V^ exhibited no significant change in LA production (**Figure 3**). We guessed the main reason for this was that both Leu and Val are non-polar hydrophobic amino acids with similar side chain groups. And also, the L243V site mutation did not significantly change the protein’s steric hindrance and surface activity. The amino acid properties (side chain structure, acidity, hydrophobicity) before and after the mutation were similar (**Supplementary Figure S4**). However, the strain transformed with PfFAD2-2^C221R^ produced LA increased to a comparable level (31.01 ± 0.13 mg/L) with those of PfFAD2-2^PF40^ (**Figure 3**). This suggests that the amino acid variation at position 221 is the primary factor contributing to the difference in catalytic ability between the LA synthases from the two perilla cultivars.

**Figure 4:**
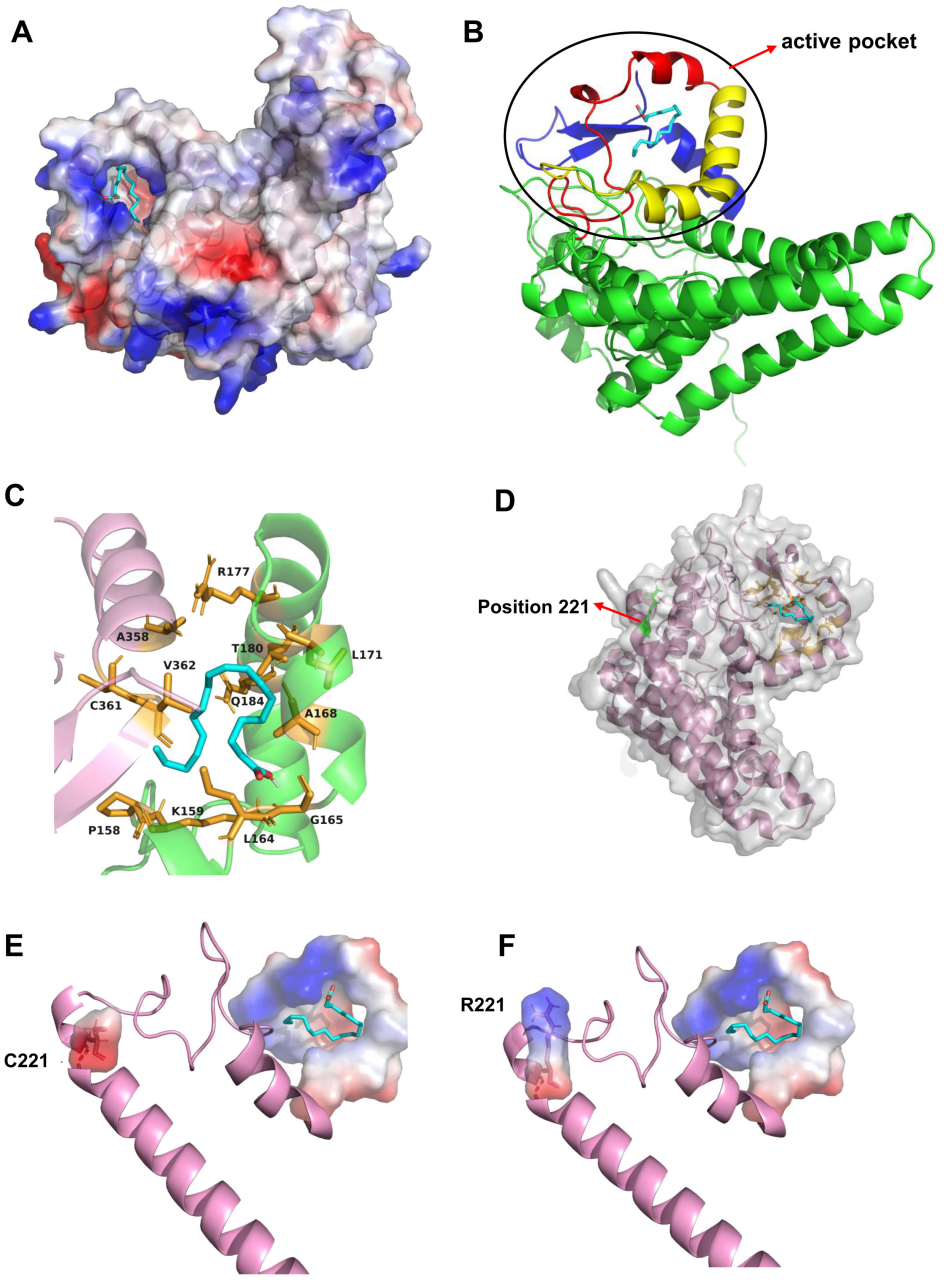
The molecular docking and residue 221 analysis of PfFAD2-2. **(A)** The docking of OA to the active pocket of PfFAD2-2. **(B)** The constitution of the active pocket of PfFAD2-2. The red represented the motif of TXSXXXDEVFVP, the yellow represented the motif of GWPXYL, and the blue represented the motif of REXXEC. **(C)** The active pocket of PfFAD2-2. **(D)** The position of residue 221 in PfFAD2-2. **(E, F)** The conformational difference of C221 and R221 in PfFAD2-2.

To further elucidate the mechanism by which the amino acid variation at position 221 affects the catalytic efficiency of PfFAD2-2, homology modeling and molecular docking were employed. Given the absence of crystal structure template for FAD2, Alphafold 2 was utilized to construct the three-dimensional structure of PfFAD2-2 (Bryant et al., 2022). Following evaluation by SAVEs 6.1 (https://saves.mbi.ucla.edu/) (**Supplementary Figure S5**), OA was docked into the protein model (**Figure 4A**). The docking analysis and multiple sequences alignment (**Supplementary Figure S6**) revealed that the active site pocket of the PfFAD2-2 structure consists primarily of the β-strand beginning with the conserved region TXSXXXDEVFVP, the α-chain terminating with the conserved region GWPXYL, and the conserved amino acid region REXXEC at the C-terminus (**Figure 4B**), and the active pocket was shown in **Figure 4C**. The amino acid at position 221 is situated on the first α-chain near the active site pocket (**Figure 4D**). Compared to the cysteine at position 221 in PfFAD2-2^PF70^ (**Figure 4E**), the arginine in PfFAD2-2^PF40^ exhibits greater rigidity, enhancing the protein’s structural stability (**Figure 4F**) (Mendes et al., 2015). Consequently, it is hypothesized that R221 in PfFAD2-2^PF40^ may augment its catalytic capability by stabilizing the protein. Additionally, homology modeling indicated that the active site pocket of PfFAD2-1^PF40^ and PfFAD2-1^PF70^ lack the conserved region REXXEC (**Figure 1A**; **Supplementary Figure S7**), which likely accounts for its lack of catalytic function.

### 3.4 Identification of additional amino acid sites affecting the catalytic ability of PfFAD2-2

**Figure 5:**
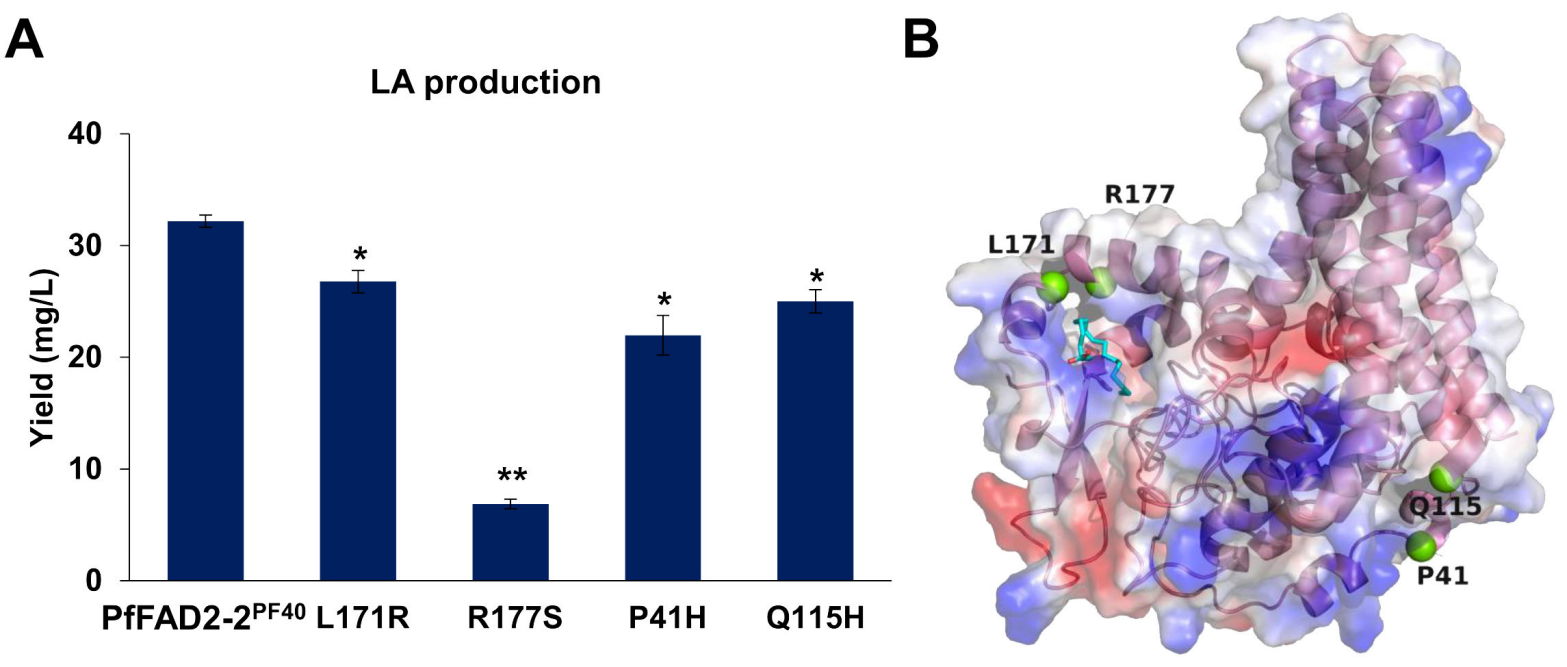
The effect of mutations in PfFAD2-2^PF40^ in LA production. **(A)** The LA production of different mutants of PfFAD2-2. All the mutants were mutated from PfFAD2-2^PF40^. The asterisks indicate significant differences (**p < 0.01, *p < 0.05). **(B)** The position of P41, Q115, L171, and R177 residues in PfFAD2-2.

Based on the above results, variations in amino acid sites were identified as the primary factors causing differences in the catalytic function of PfFAD2 among various perilla cultivars. To further elucidate the catalytic mechanism of PfFAD2, random mutation and functional verification methods were employed to identify additional amino acid sites affecting PfFAD2 catalytic efficiency. Initially, an error-prone PCR kit was utilized to amplify the *PfFAD2-2^PF40^* gene, constructing a random mutation gene library (Lu et al., 2023). These mutated *PfFAD2-2^PF40^* genes were then sequentially transformed into the engineered strain PF, followed by fermentation and LA production detection. The detection result revealed that among the thirty-two *PfFAD2-2^PF40^* mutant transformants obtained, most showed no LA production or no significant change in LA yield, with only four strains exhibiting notable yield changes. Specifically, the mutants P41H, L171R, R177S, and Q115H demonstrated reduced LA production than the WT PfFAD2-2^PF40^ (**Figure 5A**), indicating that P41, Q115, L171, and R177 are crucial residues affecting PfFAD2-2^PF40^ catalytic capacity. Subsequently, the position of these four residues were checked (**Figure 5B**) and the reasons for the decreased FAD2 catalytic efficiency caused by these mutations were hypothesized.

**Figure 6:**
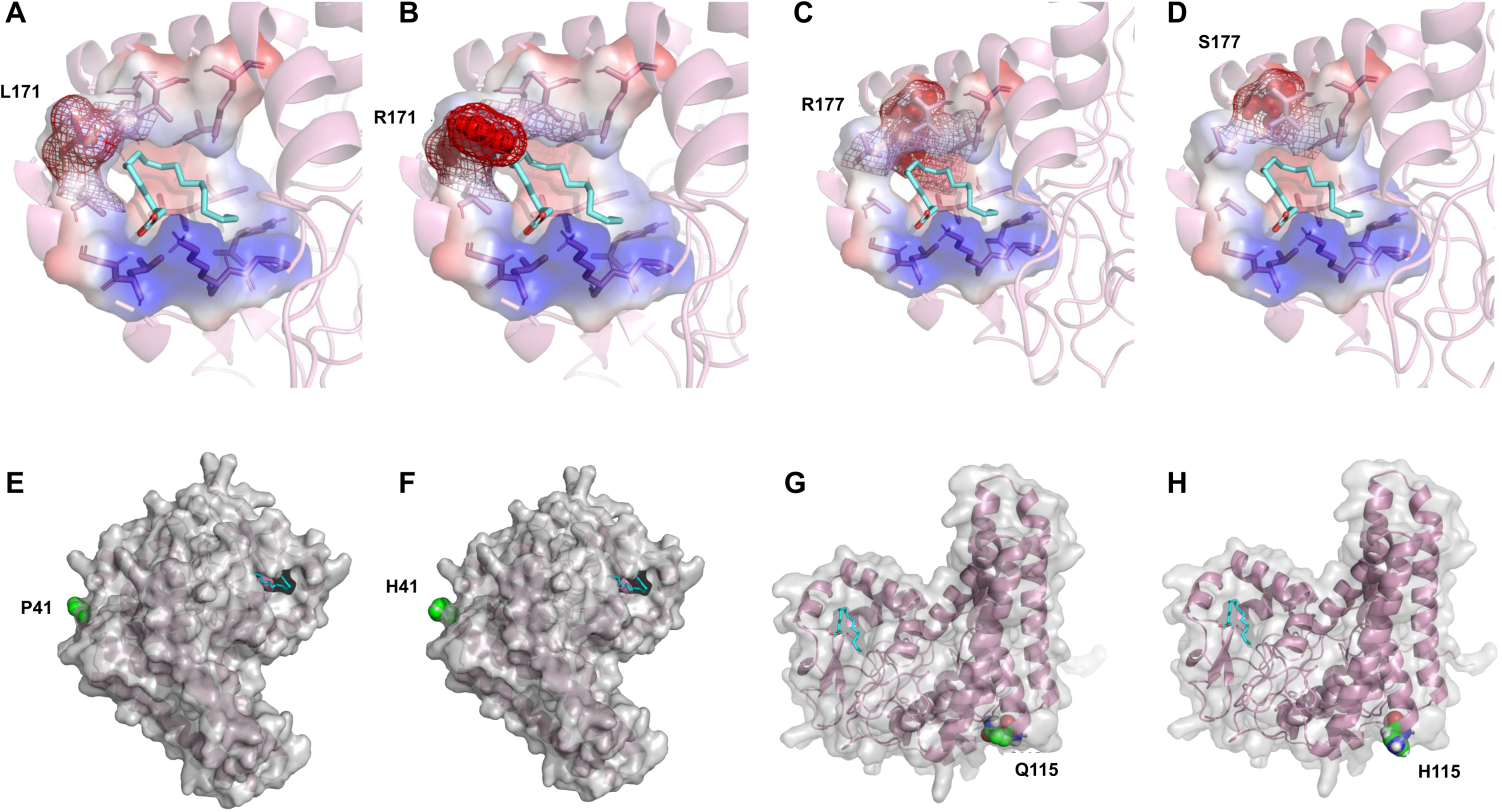
Mechanistic analysis of the catalytic activity of different PfFAD2-2 mutants. **(A–H)** Comparison of the conformational difference of L171, R177, P41 and Q115 mutants with WT.

By analyzing the distribution of these amino acid sites in the molecular docking model, it was found that L171 and R177 are located in the active pocket of PfFAD2-2^PF40^. The L171R mutation (**Figures 6A, B**) narrowed the channel entering the active pocket due to arginine having a larger side chain than leucine, thereby affecting the entry of OA molecules. The R177S mutation (**Figures 6C, D**) was unfavorable for active pocket stability, as serine has a smaller side chain than arginine. P41 (**Figures 6E, F**) and Q115 (**Figures 6G, H**) are both distributed on the enzyme surface in key loop regions connecting α-chains. The P41H mutation reduced protein stability, as histidine is less rigid than proline. The Q115H mutation decreased protein solubility, given that histidine is less hydrophilic than glutamic acid. Consequently, these mutations all adversely affected the catalytic ability of FAD2.

## 4 Discussion

Over recent decades, crop breeding research has transitioned from traditional breeding methods to molecular breeding approaches (Bai et al., 2018). Currently, the primary objectives of oilseed crop breeding encompass enhancing seed yield, quality, and stress tolerance. Specifically, increasing seed yield has evolved from genomic screening and gene editing to the application of molecular breeding techniques (Stewart-Brown et al., 2019; Anders et al., 2021; Guo, 2021). Molecular breeding emphasizes identifying trait-influencing genes, examining the relationships between these genes and known genes, and investigating the molecular mechanisms by which these genes affect traits. Advances in high-throughput sequencing, big data mining, and synthetic biology have provided valuable tools for molecular breeding (Liu et al., 2022b). In this study, *FAD2* genes were extracted from existing genomic and transcriptomic data, and their functions were verified using engineered strains. Key amino acid sites affecting PfFAD2 function were identified through sequence alignment and molecular docking analysis. This research provides a reference for the cultivation and precision breeding of perilla varieties with high LA and oil yields.

To date, FAD2 enzymes involved in LA synthesis have been identified in various organisms, including animals, plants, fungi, and algae (Jiao and Zhang, 2013). The FAD2 enzyme was initially discovered in the model plant *Arabidopsis thaliana*. It is located on the endoplasmic reticulum and catalyzes the desaturation of OA using a phospholipid-derived acyl group, with the assistance of nicotinamide adenine dinucleotide (NADH), NADH-cytochrome b5 reductase, and cytochrome b5 (Okuley et al., 1994). Differences in *FAD2* gene sequences among species and cultivars result in variations in the expression patterns and functions of LA synthases (Jiao and Zhang, 2013). Therefore, it is essential to investigate the molecular factors affecting the catalytic efficiency of LA synthases. This study explored gene variations in *FAD2* from two different perilla cultivars. However, further research is needed to understand the expression and function of other unsaturated fatty acid desaturases, such as FAD3 and FAD7, in perilla seeds (Arondel et al., 1992; Ma et al., 2015).

Amino acids form the foundation of protein structure and function. Throughout evolution, amino acids crucial to enzyme activity and stability have been conserved. Consequently, mutations at key amino acid residues can effectively alter the catalytic function of proteins (Li et al., 2024; Cai et al., 2024). For instance, manipulating the repetitive sequences of the *FAD2* gene can enhance the conversion rate of OA to LA in mouse cells (Chen et al., 2009). In this study, the conserved functional sequences of FAD2 from different perilla cultivars were compared to identify key amino acid residues responsible for variations in LA catalytic efficiency. Subsequently, homology modeling and molecular docking were employed to elucidate the structural basis for these changes in catalytic efficiency. Furthermore, to identify additional amino acid residues critical to the catalytic function of LA synthase, various FAD2-2 mutants were generated through random mutation. Functional validation and molecular docking analysis identified four important amino acid sites affecting LA production. This research provides crucial theoretical support for the genetic breeding of high oil-yielding perilla and the biosynthesis of LA.

## Data Availability

The datasets presented in this study can be found in NCBI GenBank database. The accession number(s) can be found below: PfFAD2-1^PF40^, PQ178165; PfFAD2-1^PF70^, PQ178166; PfFAD2-2^PF40^, PQ178167; PfFAD2-2^PF70^, PQ178168.
