# Supplementary material for: Crucial amino acids identified in Δ12 fatty acid desaturases related to linoleic acid production in Perilla frutescens
Source: Front Plant Sci. 2024 Sep 10;15:1464388. doi: 10.3389/fpls.2024.1464388 (PMC11420121; doi:10.3389/fpls.2024.1464388)
Supplement: Supplementary file 1 [file DataSheet1.docx]

Crucial Amino Acids Identified in ∆12 Fatty Acid Desaturases Related to Linoleic Acid Production in Perilla frutescens

# Supplementary Tables

**Table S1** The primers used in this work

| Primer Name | Sequences (5' to 3') |
| --- | --- |
| PfFAD2-F | ATGGGTGCTGGAGGGCGAA |
| PfFAD2-1-R | TTATTGGAAGACGGTGTTGAGAACGC |
| PfFAD2-2-R | CTAAAGCTTGTTATTGTACCAGAACACACCTT |
| pTP-LEU-PfFAD2-F | ACAAATATAAAACAAGCGGCCGCATGGGTGCTGGAGGGCGAA |
| pTP-LEU-PfFAD2-1-R | GTTAATTAAGAGCTCTTATTGGAAGACGGTGTTGAGAACGC |
| pTP-LEU-PfFAD2-2-R | GTTAATTAAGAGCTCCTAAAGCTTGTTATTGTACCAGAACACACCTT |
| PfFAD2-2-PF70-L243V-F | CTGTACCGTTTGACCGTTGCTAAGGGGCTCGCC |
| PfFAD2-2-PF70- L243V-R | GGCGAGCCCCTTAGCAACGGTCAAACGGTACAG |
| PfFAD2-2-PF70-C221R-F | TACTCTGACCGCGAGCGCGCTCAGATCTTCCTC |
| PfFAD2-2-PF70-C221R-R | GAGGAAGATCTGAGCGCGCTCGCGGTCAGAGTA |
| pTP-TEF1p-PGK1p-Not1-F | TCCATCGATACTAGTGCGGCCGCTTGTTTTATATTTGTTGTAAAAAGTAGATAATTACTTC |
| pTP-TEF1p-PGK1p-BamH1-R | AGTGAGTCGTATTACGGATCCTTGTAATTAAAACTTAGATTAGATTGCTATGCTTTCTT |
| pTP-URA-TGL1-Sac1-F | AGAATTGTTAATTAAGAGCTCTCATTCTTTATTTAGAGCATCCAGCGC |
| pTP-URA-TGL1-Not1-R | ACAAATATAAAACAAGCGGCCGCATGTACTTCCCCTTTTTAGGCAGATTATCG |
| pTP-URA-TGL1/5-BamH1-F | AAGTTTTAATTACAAGGATCCATGTCTAATACCTTGCCAGTAACAGAATTTCTT |
| pTP-URA-TGL1/5-Nhe1-R | TTAGAGCGGATCTTAGCTAGCTCAATTTTGAAAAATGTCTGAATTGTTCATG |
| pTP-HIS-TGL3-Sac1-F | AGAATTGTTAATTAAGAGCTCCTACCTACTCCGTCTTGCTCTTATTATGTCG |
| pTP-HIS-TGL3-Not1-R | ACAAATATAAAACAAGCGGCCGCATGAAGGAAACGGCGCAGGAATA |
| TGL1/5-ΔFAA1-F | GAGAACTTCCCTGTGCATACGGGACAGCAGAGATAGGCTGTTCTCGCGCGGGCTGGCTTAACTATGCGGCATC |
| TGL1/5-ΔFAA1-R | GCCATTTGCTTCCCATTCACCGATGTCACCGGTCTTGAACCAACCATCGCCGCGCGTTGGCCGATTCATTAATGCA |
| TGL3-ΔFAA4-F | GCTCCAACGATGTCCCCATCAATTAAGAACCCTCGATTGTCGGCGATCTAGGCTGGCTTAACTATGCGGCATC |
| TGL3-ΔFAA4-R | GAACAGATGGGTGCACCCTTAAACAGCAATTCACCTTGATTATTCTTGGCGGAAGTACCTTCAAAGAATGGGGTCTTATC |

# Supplementary Figures

**Figure S1** The schematic diagram illustrating the construction of the yeast strain

**Figure S2** The standard curves of OA (**A**) and LA (**B**)

**Figure S3** The MS spectra of LA in PfFAD2-2-transformed yeast strain (up) and LA standard (below).

**Figure S4** The comparison of 3D structures of PfFAD2-2^PF70^ and PfFAD2-2^L243V^

**Figure S5** The evaluation of homology modeling result by SAVEs 6.1. ERRAT, WHATCHECK and Ramachandran Plots indicated the reliability of the protein modeling.

**Figure S6** The multiple sequences alignment of FAD2 from different species.


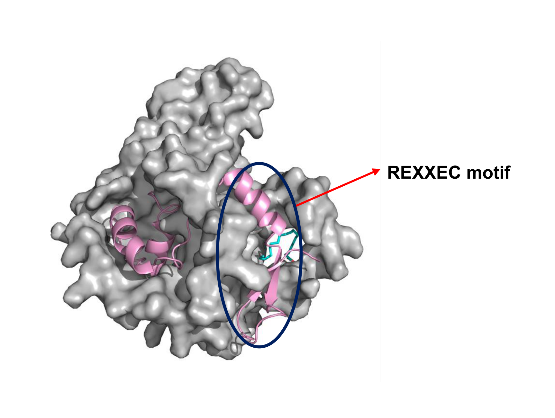


**Figure S7** The comparison of 3D structures of PfFAD2-1^PF40^ and PfFAD2-2^PF40^. The pink color represented the extra amino acid of PfFAD2-2^PF40^, and the REXXEC motif was indicated.
